# Expert consensus on relevant risk predictors for the occurrence of osteoporotic fractures in specific clinical subgroups – Delphi survey

**DOI:** 10.1186/s41927-019-0099-y

**Published:** 2019-11-12

**Authors:** Nicolas S. Bodmer, Hans Jörg Häuselmann, Diana Frey, Daniel Aeberli, Lucas M. Bachmann

**Affiliations:** 1 Medignition Inc. Medical Research Consultants, Verena Conzett-Strasse 9, 8004 Zurich, Switzerland; 2 Center for Rheumatology and Bone Disease, Bellariastrasse 38, 8038 Zurich, Switzerland; 3 Division of Rheumatology, University Hospital Zurich, Gloriastrasse 25, 8091 Zurich, Switzerland; 4 Department of Rheumatology and Clinical Immunology/Allergology, University Hospital Bern, Freiburgstrasse 18, 3010 Bern, Switzerland; 5 University of Zurich, Rämistrasse 71, 8006 Zurich, Switzerland

**Keywords:** Primary osteoporosis, Secondary osteoporosis, Fracture prediction, Older adults, Expert consensus, Delphi consensus method

## Abstract

**Background:**

There is an ongoing discussion about incorporating additional risk factors to established WHO fracture risk assessment tool (FRAX) to improve the prediction accuracy in clinical subgroups. We aimed to reach an expert consensus on possible additional predictive parameters for specific clinical subgroups.

**Methods:**

Two-round modified Delphi survey: We generated a shortlist of experts from the authors’ lists of the pertinent literature and complemented the list with experts known to the authors. Participants were asked to name possible relevant risk factors besides the FRAX-parameters for the occurrence of osteoporotic fractures. Experts specified these possible predictors for specific subgroups of patients. In the second round the expert panel was asked to weight each parameter of every subgroup assigning a number between one (not important) to ten (very important). We defined the threshold for an expert consensus if the interquartile range (IQR) of a predictor was ≤2. The cut-off value of the median attributed weights for a relevant predictor was set at ≥7.

**Results:**

Eleven experts of seven countries completed both rounds of the Delphi. The participants agreed on nine additional parameters for seven categories. For the category “secondary osteoporosis”, “older adults” and “nursing home patients”, there was a consensus that history of previous falls was relevant, while for men and postmenopausal women, there was a consensus that the spine fracture status was important. For the group “primary and secondary osteoporosis” the experts agreed on the parameters “high risk of falls”, “lumbar spine bone mineral density (BMD)” and “sarcopenia”.

**Conclusion:**

This Delphi survey reached a consensus on various parameters that could be used to refine the currently existing FRAX for specific clinical situations or patient groups. The results may be useful for studies aiming at improving the predictive properties of instruments for fracture prediction.

## Background

Today fracture prediction is made with the well-established WHO fracture risk assessment tool (FRAX), but there is still a discussion on the completeness of the tool in terms of included parameters particularly for clinical subgroups. So far, there is no international expert consensus of fracture prediction parameters which impedes adequate therapy decisions. FRAX is based on individual patient models that integrate the risks associate with eleven, mostly binary, clinical risk factors and bone mineral density [1]. Incorporating additional parameters as well as adaptations of predictors are under consideration by osteoporosis experts aiming at an optimized fracture prediction. For instance, a previous unspecific fracture is classified as a risk factor by the WHO tool. This potentially includes low trauma metatarsal fracture, although it is questionable whether fractures of the hand and foot are predictors at all [2]. Additional parameters such as vitamin D-levels, falls or balance measures are under discussion for enhancing accurate fracture prediction [2, 3]. Several researchers raised the question, whether early identification of higher fracture risk could be improved.

We aimed to reach a consensus among international experts regarding possible additional parameters besides the well-established FRAX parameters in different treatment subgroups using a two round modified Delphi.

## Methods

We conducted a two-round modified online Delphi survey. The Delphi method is a systematic approach to obtain opinions of a group of experts by means of a series of short self-administered questionnaires [4].

### Ethics and informed consent

No formal ethics approval was necessary for this study. Participants received a full written explanation about the scope and goals of the study. All experts participated voluntarily and provided written consent by completing the two Delphi rounds.

### Identification of panel members

One researcher generated a list of potential participants of the expert consensus panel. Candidates were identified by checking the author lists of primary studies summarized in a recent systematic review [5]. Contact details of the first, second and last author of each publication were retrieved from the original publication. We complemented the list with experts known to the authors. Current e-mail addresses were verified via a general web search. Fifty experts were invited to participate. Every expert received an e-mail containing a description of the purpose of the study and an invitation to participate. Anonymous participation was granted to ensure freedom of expression. If an e-mail was rejected, we contacted the last known host institution of the expert. A reminder letter was sent out two weeks later.

### Survey set-up

The questionnaire of the first round was designed by one research fellow. Following the approval from all authors the questionnaire was implemented into the survey software. The survey was conducted using the SurveyMonkey online tool [6]. The online tool allows to implement an introduction page providing information about the survey methods. Also, the system dynamically adapts its user interface offering sufficient space for the number of answers given. Moreover, it is possible to set input requirements allowing only a specific format of the answer e.g. range of the attributed weights must be a number between one and ten.

### First round questionnaire

Each consenting expert received a personalized e-mail containing a description of the study method and a web-link to access the survey. Before starting to complete the questionnaire, each participant provided information about his or her specific expertise and the current affiliation. In the first round, experts were asked to name possible relevant predictors besides the well-established parameters of FRAX for the occurrence of osteoporotic fractures. They could specify these possible predictors for one or more specific subgroups of patients (e.g. patients with secondary osteoporosis).

### Second round questionnaire

The feedback from the first round was extracted, examined and prepared for the second round. Obvious misspellings were corrected and plurals were replaced by singulars (e.g. fall vs. falls). Terms with the same root word were collapsed into one term if the intended meaning remained unchanged. If appropriate, a general concept was used to summarize a specific condition (i.e. kidney transplantation, lung transplantation, heart transplantation was collapsed into one term “history of transplantation”). In the questionnaire, the parameters were then grouped into the seven different subgroups. Every participant received after round one a compiled inventory of the answers. In the second round, participants weighed for each subgroup every predictor by assigning a number between one (not important) and ten (very important).

### Attaining a consensus on relevant parameters

We calculated the median of the attributed weights and the corresponding interquartile ranges (IQR) for each of the parameters within each of the clinical subgroups. Using the thresholds of a previous Delphi study, we defined an expert consensus as the IQR of a predictor ≤2 [7]. The cut-off value of the median attributed weights for a relevant predictor was set at ≥7. Statistical analyses were performed using the Stata 14.2 statistical software package (StataCorp. 2015. Stata Statistical Software: Release 14. College Station, TX: StataCorp LP.). At the end of the Delphi study, every participant received the results of the Delphi including median and IQR of all items.

## Results

Eleven experts from Australia [1], Canada [1], France [1], Israel [1], Italy [1], Norway [1], and USA [5] participated in the survey. The first round revealed thirty-six parameters for seven clinical subgroups, older adults, postmenopausal women, men, secondary osteoporosis, primary and secondary osteoporosis, nursing home patients and patients close to treatment threshold. Table 1 shows the attributed median weight of the thirty-six parameters and the corresponding IQR in descending order.

**Table 1:** Predictors with attributed median weight and corresponding IQR

Patient subgroups	Predictor	Median	IQR
*Secondary osteoporosis*			
	**recent or multiple falls**	9	1
	aromatase inhibitors	6.5	1
	chronic obstructive pulmonary disease	6.5	2
	history of transplantation	6.5	2.5
	decrease in BMD over the previous 2 years	6.5	2.5
	androgen deprivation therapy (prostate cancer)	6.5	2.5
	rheumatic diseases other than rheumatoid arthritis (excluded osteoarthritis)	6.5	3
	breast cancer therapy	6	1
	inflammatory activity of the underlying disease	6	3
	severe disability form multiple sclerosis or other neurodegenerative conditions	6	3
	Crohn’s disease	5.5	1.5
	low grip strength	5.5	1.5
	gastric bypass surgery	5.5	2.5
	HIV	5	1.5
*Primary and secondary osteoporosis*			
	**high risk of falls**	9.5	1
	**lumbar spine BMD**	7.5	1.5
	**sarcopenia**	7	1.5
	dismobility syndrome	6	2
*Postmenopausal women*			
	**spine fracture status**	10	0.5
	major height loss	8	3.5
	kyphosis	6.5	1.5
	early surgical menopause	6	2.5
*Nursing home patients*			
	**falls**	10	1.5
	functional status	8	3
*Men*			
	**spine fracture status**	10	0.5
	hypogonadism	4.5	3.5
*Older adults*			
	**falls**	9.5	2
	functional/ambulatory status	8.5	4.5
	medications (particularly psychotropic medications)	8	3
	stroke	6	2
	type 2 diabetes	6	2
	Parkinson’s disease	6	2.5
*Close to treatment threshold*			
	**lumbar spine BMD (for vertebral fracture prediction)**	8	1.5
	type 2 diabetes	6	3.5
	trabecular bone score (TBS)	5.5	3.5
	hip axis length (for hip fracture prediction)	4	4

Predictors fulfilling the consensus criteria are marked bold

The participating experts agreed on nine additional parameters for the seven categories. The final set of predictors is shown in Table 2. For the category “secondary osteoporosis”, “older adults” and “nursing home patients”, there was a consensus that previous falls were relevant, while for men and postmenopausal women, there was a consensus that the spine fracture status was important. For the group “primary and secondary osteoporosis” the experts agreed on the parameters “high risk of falls”, “lumbar spine bone mineral density (BMD)” and “sarcopenia”. For the group close to the treatment threshold, “lumbar spine BMD” was considered relevant as a predictor for vertebral fracture prediction.

**Table 2:** Consensus of relevant fracture predictors

Patient subgroups	Predictor	Median	IQR
*secondary osteoporosis*	recent or multiple falls	9	1
*primary and secondary osteoporosis*	high risk of falls	9.5	1
	lumbar spine BMD	7.5	1.5
	sarcopenia	7	1.5
*postmenopausal women*	spine fracture status	10	0.5
*nursing home patients*	falls	10	1.5
*men*	spine fracture status	10	0.5
*older adults*	falls	9.5	2
*close to treatment threshold*	lumbar spine BMD (for vertebral fracture prediction)	8	1.5

The participants of the expert panel disagreed on four parameters with a high median (≥7) but IQR > 2. Namely the parameter “major height loss” of the subgroup “postmenopausal women”, “functional status” of the subgroup “nursing home patients”, “functional/ambulatory status” and “medications particularly psychotropic medications” of the subgroup “older adults”.

## Discussion

### Main findings

In this Delphi survey, a group of international experts agreed on additional risk parameters for osteoporotic fractures currently not covered in the WHO FRAX instrument and converged on seven different subgroups needing specific assessments. The expert consensus was highest on the predictor “recent falls” for the occurrence of osteoporotic fracture followed by spine fracture status or lumbar spine (bone mineral density (BMD)) depending on the subgroup. The parameters of clinical history “HIV”, “Crohn’s Disease” and the item “low grip strength” were considered less important for fracture prediction. Surprisingly, no consensus could be reached for parameters such as “functional / ambulatory status” in older adults.

### Results in context of the existing literature

We are unware of any previous Delphi consensus on fracture predictors beside the established FRAX parameters. In a recent systematic review summarizing 45 studies, the authors compared thirteen fracture prediction tools and included 20 of the 45 studies in the meta-analysis grouped into three categories. In women without a BMD result available at the time point of the risk assessment, the FRAX tool was less accurate than the QFracture tool, another fracture prediction instrument published in 2009. If BMD results were available when assessing the patient, the FRAX instrument was more predictive than the QFracture tool [8, 9]. QFracture records additional predictors such as systemic lupus erythematosus (SLE), gastrointestinal malabsorption, anticonvulsants, dementia or history of falls which indicates the need for reconsidering the set of used predictors. History of falls was also considered very relevant for the experts of the survey, while other predictors of the QFracture tool were not. The results of the Delphi Study are consistent with previous studies which identified falls in older adults as an independent predictor for the occurrence of fractures [2, 10, 11].

The FRAX model uses a dichotomized variable for the risk factor “previous fracture” which could lead to an underestimation of fracture risk as a result of the progressively increasing risk with the number of prevalent vertebral fractures [12, 13]. Furthermore, the severity and type of fracture is pivotal for risk assessment as a prior vertebral fracture had a lower prognostic value for further non-vertebral fractures than for vertebral fracture outcomes [14].

### Strength and limitation

A strength of the Delphi study is the inclusion of geographically dispersed experts and the avoidance of undue dominance by individuals through anonymity [15]. It could be argued that the small number of experts contributing to this survey limits the extent to which results can be generalized. However, this would predominately affect the first round of the Delphi and not necessarily the consensus made in round 2. Moreover, all experts had many years of experience in their field and it can be assumed that they covered the theoretical and practical aspects of osteoporosis management thoroughly. Finally, the thresholds set for relevance and consensus are somewhat arbitrary. We cannot fully rule-out that setting these thresholds differently would affect the overall message of this paper to some extent. By providing the full list of replies along with the medians and the interquartile ranges we offer readers the possibility to assess the consequences if different thresholds would have been chosen.

### Implications for practice and further research

This paper presents a set of agreed parameters besides the FRAX predictors that experts considered as relevant for enhancing accurate fracture prediction. On one hand, the evaluated predictors may help clinicians in the risk assessment of the individual patient. On the other hand, we provide a basis for further discussions about possible additional fracture predictors. In an ongoing study of our research group we incorporated the parameters found in this consensus study. This will allow us to quantify improvements in the prediction when using these additional parameters in the pre-specified subgroups of patients. Further research should aim to assess the reported predictors with clinical data to confirm the importance of the items. Also, new or modified existing fracture assessment tools with additional predictors should be validated and compared with current practice. This study could guide the direction of further research and developing efforts regarding the ongoing discussion about risk factors for osteoporotic fractures.

## Conclusions

In this Delphi survey, an international group of experts reached a consensus on various parameters that could be used to refine the currently existing FRAX tool for specific clinical situations or patient groups. The results may be useful for studies aiming at improving the predictive properties of instruments for fracture prediction.

## Data Availability

The datasets used and/or analysed during the current study are available from the corresponding author on reasonable request.

## Abbreviations

BMD: Bone mineral density
FRAX: WHO fracture risk assessment tool
IQR: Interquartile range
SLE: Systemic lupus erythematosus
